# Novel Use of Meniscal Allograft Arthroplasty for Basal Joint Arthritis in a Patient With Ehlers-Danlos Syndrome: A Case Report

**DOI:** 10.7759/cureus.98522

**Published:** 2025-12-05

**Authors:** Lucas Vieira, Emma M Eng, Ritesh Chandrasekaran, Ehsan Esmaeili

**Affiliations:** 1 College of Medicine, Florida Atlantic University Charles E. Schmidt College of Medicine, Boca Raton, USA; 2 Department of Orthopaedic Surgery, Hand and Ortho, Boca Raton, USA; 3 Department of Orthopaedics, Florida Atlantic University Charles E. Schmidt College of Medicine, Boca Raton, USA

**Keywords:** connective tissue disorder, ehlers danlos syndrome, meniscal allograft, meniscal allograft arthroplasty, orthopaedic hand surgery, thumb basal joint arthroplasties, thumb carpometacarpal arthritis, thumb carpometacarpal joint osteoarthritis

## Abstract

Ehlers-Danlos Syndrome (EDS) is a complex condition to treat surgically due to the nature of the disease. Thumb carpometacarpal (CMC) arthritis is a particularly prevalent condition that often requires surgical intervention for symptomatic and motor relief. A 56-year-old female patient with EDS opted for surgical treatment after persistent pain of the joint refractory to conservative treatment after one year. With no complications and an unremarkable recovery period, the patient has returned to baseline function with no restrictions in activities of daily living, significantly reduced reported pain, and a high level of reported satisfaction with the results of this procedure at six-month postoperative follow-up. This case report aims to highlight the outcomes of a possible treatment option for CMC arthritis in patients with EDS using meniscal allograft arthroplasty.

## Introduction

Ehler-Danlos Syndrome (EDS) is a heterogeneous connective tissue disorder affecting collagen synthesis. The impaired collagen synthesis leads to skin hyperextensibility, tissue fragility, and joint hypermobility [1]. Multiple tissue types are impacted in this condition, including the skin, ligaments, tendons, and cartilage [2]. EDS can often be challenging to diagnose due to ambiguity in clinical presentation and lack of definitive diagnostic testing [1]. Due to joint defects, patients with EDS often develop basal joint arthritis, also known as carpometacarpal (CMC) arthritis. Therefore, it is critical that physicians understand the variation in presentation of the condition and recognize the risks of developing chronic joint issues [3].

Physicians will first attempt non-operative treatment for CMC arthritis prior to opting for surgical intervention. Conservative approaches consist of “R-I-C-E” (Rest, Ice, Compression, Elevation), anti-inflammatory analgesics, physical therapy, and splints. The efficacy of non-operative treatment varies and is highly dependent on the location and the severity of a patient’s symptoms [1]. After failing non-operative treatment, patients in one study demonstrated decreased pain levels, increased range of motion (ROM), and improved grip strength in this patient population following CMC allograft arthroplasty and trapeziectomy [4]. Despite multiple surgical options, it remains paramount to understand the risks associated with EDS in an operative setting, including recurrent ligament instability, worsening postoperative pain levels, infection, and failed intervention [1,5]. Meniscal allograft arthroplasty as a means of treating CMC arthritis remains sparsely investigated, and the use of this procedure has not yet been reported in an EDS patient. The aim of this case report is to present the potential use and demonstrated results of meniscal allograft arthroplasty in treating CMC arthritis in patients with EDS. Informed consent for publication was obtained from the patient in accordance with the CARE guidelines.

## Case presentation

**Figure 1:**
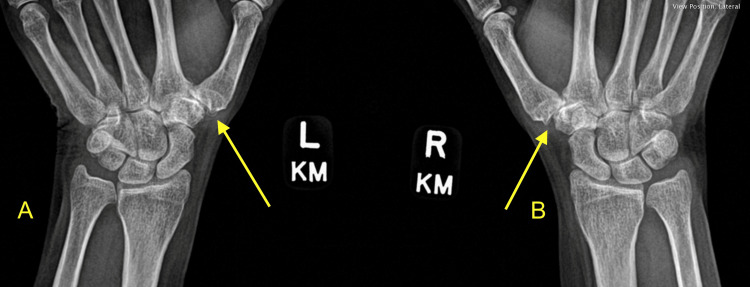
X-ray displaying preoperative degenerative changes of bilateral CMC joints. (A) Left CMC joint arthritis with arrow indicating the joint space narrowing, osteophyte formation, and joint subluxation. (B) Right CMC joint arthritis with arrow indicating joint space narrowing, osteophyte formation, and joint subluxation. CMC: carpometacarpal

A 56-year-old female patient first presented in October 2023 with bilateral thumb pain. Her past medical history was remarkable for diagnoses of rheumatoid arthritis and EDS, Type III. Upon primary survey of the patient’s upper extremities, synovitis was noted in the CMC thumb joints bilaterally. In addition, there was significant localized pain and weakness noted with active pinch, and a positive CMC grind test was rendered bilaterally. Examination of the wrists and elbows was unremarkable. Bilateral basal joint radiographs showed degenerative changes (Figure 1).

These changes include significant joint space narrowing with metacarpal subluxation of the trapezium, arthritic scaphotrapeziotrapezoidal (STT) joints, and sclerosis and osteophyte formation in the subchondral space. The patient initially elected to continue with conservative therapy and received a cortisone injection with the addition of an opponens splint. The patient then returned twice, in December 2023 and March 2024. At these times, she had an unchanged complaint of pain after receiving minimal relief from the injection.

After a year of conservative treatment, in October 2024, the patient elected to undergo a right CMC arthroplasty using an ALLIO meniscal allograft (A.M. Surgical Inc., Smithtown, New York, United States). The procedure was completed in December 2024 with no intraoperative or postoperative complications. The hand surgeon had developed a novel means of treating CMC joint arthritis with a meniscal allograft arthroplasty called the “Taco Technique”. With promising outcomes in other patients, the surgeon was comfortable treating patients with EDS.

Surgical technique

The technique involved creating a 5-6 cm incision over the thumb CMC joint in order to expose the joint. Careful dissection of the subcutaneous tissues and the capsule was carried out down to the capsule, where a 2-3 mm wafer of bone was sawed off the base of the metacarpal and trapezium to allow space for the allograft. The base of the thumb metacarpal and trapezium was drilled at three points: 9, 12, and 3 o’clock, for securing of the graft. In preparation for allograft insertion, the graft was folded in a taco-like configuration and secured with 3.0 Ethibond sutures (Ethicon Inc., Raritan, New Jersey, United States) via the midportion of the graft through the drilled holes and back through the midportion for tying. A 4.0 Supramed suture (S. Jackson, Inc., Alexandria, Virginia, United States) was then passed through the drill holes to accommodate fixation of the graft. The tissue was then closed layer by layer with 3.0 Vicryl (Ethicon Inc.), carefully, until reaching the skin. Hemostasis was achieved, and the skin was closed with a 5.0 nylon suture. A sterile dressing was carefully applied to the incision site, and this completed the procedure. The thumb was then immobilized with a thumb spica splint. This approach does not require permanent manipulation of the ligaments or tendons surrounding the joint.

Postoperative management

**Figure 2:**
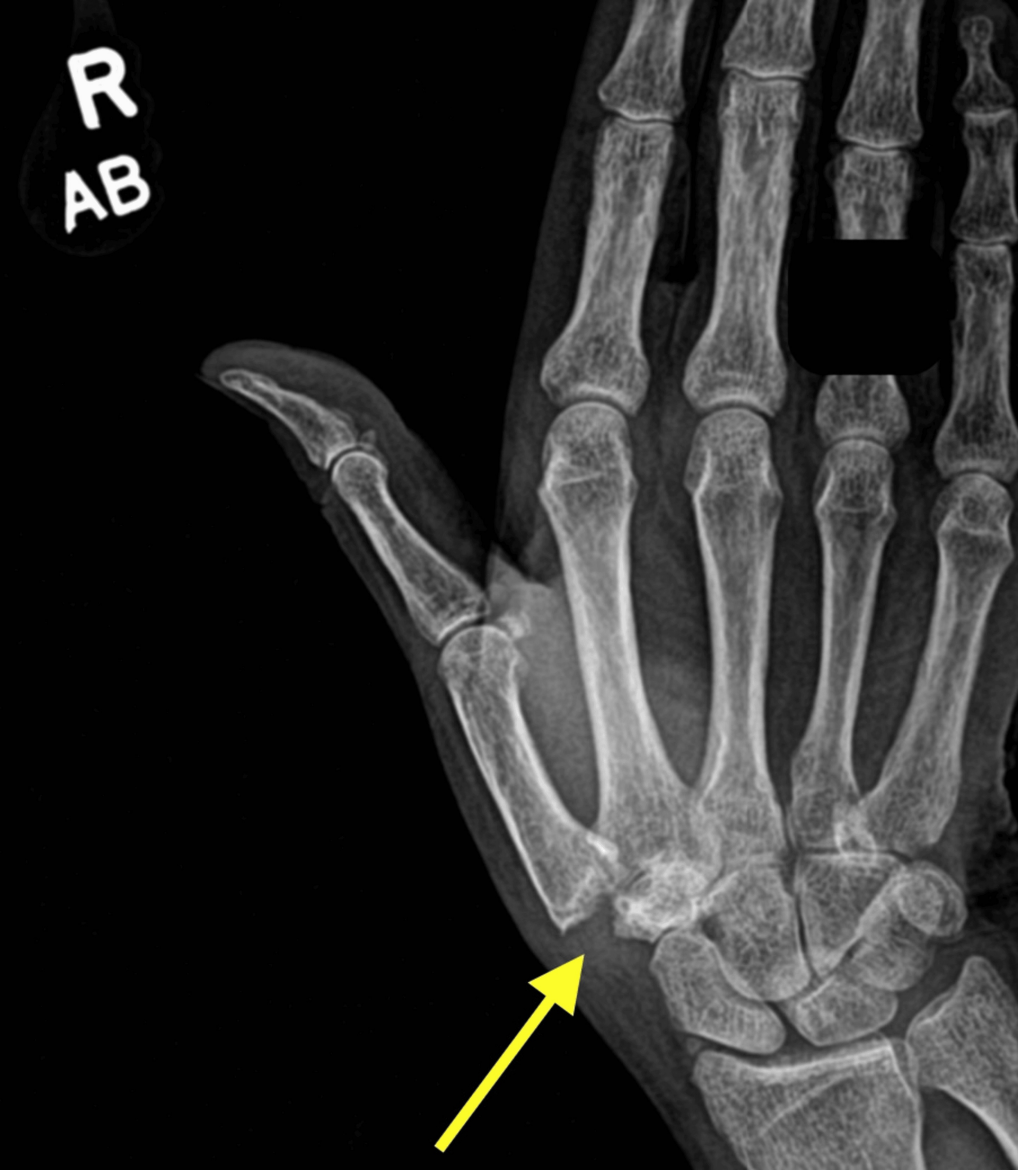
Postoperative imaging of the right CMC joint indicated by the arrow CMC: carpometacarpal

The patient returned to the office one week later for her first postoperative follow-up. The incision was healing well, and there were no signs of infection or cellulitis. A thumb spica splint was ordered to be worn 24 hours a day until the next follow-up visit in January 2025. At this visit, the wound was continuing to heal without issue. She was allowed to remove her thumb brace and began occupational therapy three times a week, in order to encourage motion recovery of the thumb. The patient had one last postoperative follow-up visit in February 2025, where there were no issues and satisfactory recovery in motion with her therapies. She was discharged and instructed to follow up as needed. Her last imaging of the right CMC joint was taken in April 2025 (Figure 2).

Upon telephone follow-up at her six-month postoperative mark, the patient reported great satisfaction with the results of her surgery. The patient denied any use of pain medication and reported a rare occurrence of pain, as little as once a month. In the event that she did have pain, she reported it was mild, ranked a 2/10 on a Likert scale.

The patient no longer needs to attend regular occupational therapy sessions, though she incorporates her stretches to ease the rare episodes of pain. Her reported range of motion has been restored to preoperative levels, and she is able to perform all her activities of daily living without restrictions. She will consider the same basal joint reconstruction technique if her left joint worsens clinically. Overall, this patient reports no issues with any part of the surgical and recovery processes and would confidently recommend this procedure to other patients presenting with similar complaints.

## Discussion

It is challenging to treat patients with EDS surgically for many reasons. A specific challenge in the case of musculoskeletal pathology is joint hypermobility and ligamentous instability [4,6,7]. Due to the widespread connective tissue weakness in these patients, joint reconstruction surgeries have been generally discouraged. Not only is the surgical plan difficult to create due to the challenging anatomy, but the postoperative complications also remain an area of concern. A recent systematic review demonstrated that patients with EDS who underwent an orthopedic procedure suffered from high rates of persistent pain, joint instability, and repeated joint dislocation [5]. In contrast, following the use of the demonstrated novel technique utilizing meniscal allograft arthroplasty, results of this case have thus far been favorable, with rarely reported episodes of pain, a full return to baseline function within six months postoperatively, and no continued need for occupational therapy. Additionally, as there is no current evidence-based research to guide surgical treatment in patients with EDS [8], the “Taco Technique” offers an innovative and novel surgical approach for potential use in this patient population.

Case reports citing joint surgical treatment for patients with EDS are uncommon, and the recommendations for these patients are not thoroughly explored. A study by Hevesi et al. examined a total of nine patients with both EDS and CMC arthritis and compared operative and nonoperative therapy results to evaluate the effectiveness of both [4]. The nonoperative group was managed with non-steroidal anti-inflammatory drugs (NSAIDs), splints, and occasional steroid injections. No significant change in grip strength, oppositional pinch, or appositional pinch was found between the first and last follow-up. However, there was an observed decreasing trend in the ROM of the joint over time. Pain did not significantly change. On the other hand, the operative group experienced a significant reduction in pain following surgical treatment and increased grip strength over time. However, there was no significant difference in the strength of pre- and postoperative grip strength, oppositional pinch, or appositional pinch. This sample underwent different operations, including metacarpophalangeal fusion with trapeziectomy and suspension plasty using a plantaris allograft for one patient. A second patient underwent a bilateral CMC arthroscopy and synovectomy with capsular shrinkage. The third operative patient underwent a CMC hemiarthroplasty, later revised with trapeziectomy and CMC arthroplasty with gracilis allograft. At a later date, a plantaris allograft was used for the opposite side. The last operative patient underwent trapeziectomy and CMC arthroplasty with flexor carpi radialis autograft. This study suggests that operative management of CMC arthritis may provide greater increases in returned strength with significant reductions in pain in this patient population [4]. However, it should be noted that the study [4] only followed patients who underwent ligament reconstructions in addition to trapeziectomy, which differs from the current case’s reported surgical approach of a meniscal allograft arthroplasty without requiring ligament manipulation.

Trapezial opening wedge osteotomy and volar ligament reconstruction have previously been used for the surgical treatment of an 18-year-old female patient with EDS who presented with joint instability [9]. The procedure was described as a success as the patient was reported to be pain-free with joint stability and an 18 kg improvement in grip strength at a two-year postoperative follow-up. An earlier case report shared the results of a 56-year-old male patient with CMC arthritis managed with bilateral tendon interposition arthroplasty coupled with thermal capsular shrinkage [10]. At the two-month postoperative follow-up, there was reported excellent pain relief in both joints. One year following surgical intervention, the patient reported no pain and returned to normal activity. The patient demonstrated improved grip and pinch strengths compared to the preoperative baseline. A final case report explored the results of a 58-year-old female patient with suspected EDS with bilateral CMC arthritis who was treated with CMC arthroplasty and ulnar collateral ligament reconstruction of the left thumb and later underwent CMC arthroplasty of the right thumb [7]. The recovery period was uneventful with minimal reported pain, well-healing incisions, and a near full ROM reported by the six-week postoperative visit. This patient reported a high level of satisfaction with the procedure results at the three-month postoperative visit. At this point in time, only three studies have been published investigating surgical treatment of CMC arthritis and outcomes in patients with EDS (Table 1) [4,7,10].

**Table 1:** Operative treatment outcomes for carpometacarpal arthritis in patients with Ehlers-Danlos Syndrome CMC: carpometacarpal; EDS: Ehlers-Danlos syndrome; HLA: human leukocyte antigen

Authors (Year)	Title	Patient Presentation	Surgery	Outcomes
Badia et al. 2005 [10]	Bilateral arthroscopic tendon interposition arthroplasty of the thumb carpometacarpal joint in a patient with Ehlers-Danlos syndrome	56-year-old male with bilateral CMC arthritis and EDS	Bilateral arthroscopic tendon-interposition arthroplasty of the thumb carpometacarpal joint with thermal capsular shrinkage	The patient sustained excellent pain relief in both thumbs at two months postoperatively. At one year postoperatively, the patient reported no pain and a full return to function. The patient demonstrated improved grip and pinch strengths compared to the preoperative baseline.
Hevesi et al. 2019 [4]	Thumb carpometacarpal arthritis in patients with Ehlers-Danlos Syndrome: non-operative and operative experiences	Four patients with CMC arthritis and coincident EDS syndrome were managed operatively. One 61-year-old female with right-sided CMC arthritis and laxity, one 41-year-old male, one 64-year-old female with bilateral CMC arthritis, and one 52-year-old male with bilateral CMC arthritis and concurrent HLA B-27 spondyloarthropathy	Respective surgical approaches included: metacarpal fusion with trapeziectomy and suspensionplasty with a plantaris allograft, bilateral staged first CMC arthroscopy with synovectomy and capsular shrinkage spaced four months apart, left pyrocarbon first CMC hemiarthroplasty and first CMC arthroplasty with gracilis allograft with hemiarthroplasty revision at 34 months from pain and later performance on the right side with plantaris allograft, and lastly trapeziectomy and first CMC arthroplasty with flexor carpi radialis autograft	Respective outcomes are as follows: no pain with occasional weightlifting at the 1.5-year follow-up, improved pain and working as part-time dentist at the 15-year follow-up, ability to participate in activities with occasional pain at the 8-year follow-up, and lastly mild to no symptoms reported at the 4.5-year follow-up with return to work at 6 months postoperatively.
Samona and Palazzo, 2019 [7]	Carpometacarpal arthroplasty and ulnar collateral ligament reconstruction in a patient with suspected Ehlers-Danlos syndrome: a case report and review of the literature	A 58-year-old woman with bilateral thumb CMC arthritis and metacarpophalangeal joint hypermobility, suspected to be EDS	CMC arthroplasty with ulnar collateral ligament reconstruction of the left thumb	Minimal reports of pain postoperatively with no complications. Nearly full range of motion regained by her six-week postoperative visit. At the three-month follow-up, she reported high levels of satisfaction with her outcome. Right CMC arthroplasty was performed one month later with similar satisfying recovery with no complications and improved strength and function of the left thumb.

From the success of this individual case, this operative approach may be considered as a potentially effective and safe procedure for this patient population or others of similar connective tissue conditions. However, while the results of this case are promising, future studies should be conducted with a larger sample size, comparative outcomes with other surgical approaches, and longer follow-up duration in order to properly support the use of this surgical technique.

## Conclusions

While EDS creates many challenges when treating joint pathologies, the results of this case show that CMC arthritis in this patient population may be successfully treated with surgical intervention, specifically the one highlighted in this report. We presented a novel surgical approach, the “Taco Technique”, which utilizes a meniscal allograft for CMC arthroplasty with encouraging results. This report highlighted the potential of this approach in successfully treating CMC arthritis in this patient population and proposes an option for hand surgeons. Additionally, this technique does not involve ligament manipulation and may provide an earlier operative option, leaving room to progress to ligament reconstruction if symptoms remain refractory.

This case report adds to the growing literature of effective operative treatments for CMC arthritis in patients with EDS. Future research with larger sample sizes and control groups is necessary to produce generalizable results utilizing this approach and better establish recommendations for the most effective operative treatment options in this patient population.
